# Preoperative angiotensin-converting enzyme inhibitor or angiotensin receptor blocker use and acute kidney injury after high-risk cardiac surgery: a multicenter prospective cohort study^1^

**DOI:** 10.1016/j.bjane.2026.844786

**Published:** 2026-07-01

**Authors:** Iñigo Rubio, Angel Candela, Gemma Echarri, Raquel Callejas, Eduardo Tamayo, David Nagore, Jorge M. Núñez-Córdoba, Marc Vives

**Affiliations:** a Clínica Universidad de Navarra, Department of Anesthesiology & Critical Care Medicine, Pamplona, Spain; b Hospital Universitario Ramón y Cajal, Department of Anesthesiology & Perioperative Medicine, Madrid, Spain; c Hospital Clínico Universitario de Valladolid, Department of Anesthesiology & Perioperative Medicine, Valladolid, Spain; d St Bartholomew's Hospital, Barts Heart Centre, Department of Anaesthesia & Perioperative Medicine, London, United Kingdom; e Clínica Universidad de Navarra, Central Clinical Trials Unit, Research Support Service, Pamplona, Spain; f Public University of Navarra, Department of Health Sciences, Pamplona, Spain; g Instituto de Investigación Sanitaria de Navarra, IdiSNA, Pamplona, Spain

**Keywords:** Acute kidney injury, Angiotensin-converting enzyme inhibitors, Angiotensin receptor antagonists, Cardiac surgical procedures, Kidney diseases, Risk factors

## Abstract

**Background:**

The impact of preoperative Angiotensin-Converting Enzyme Inhibitors or Angiotensin Receptor Blockers (ACEI/ARB) on cardiac surgery-associated Acute Kidney Injury (AKI) remains elusive. We sought to evaluate the association of ACEI/ARB use with stage 2 or 3 AKI and in-hospital mortality, while evaluating whether preoperative withholding versus continuation strategies modulate clinical outcomes.

**Methods:**

Multicenter prospective cohort study involving 14 Spanish and British hospitals. The study population comprised high-risk cardiac surgery patients (Cleveland Clinic Score ≥ 4).

**Results:**

Among 249 patients (mean age: 69.5 years; 39% women; 53.8% ACEI/ARB users), the overall incidence of stage 2 or 3 AKI was 32.9%. ACEI/ARB use was associated with a significantly higher likelihood of stage 2 to 3 AKI (38.8% vs. 26.1%; adjusted OR = 2.79; 95% CI 1.47–5.30; p = 0.002). The adjusted risk difference was 0.188 (95% CI 0.079–0.298; p = 0.001), yielding a number needed to harm of 5.3. Sensitivity analyses confirmed the robustness of these findings, with an E-value of 2.73 (lower limit: 1.72) and consistent results using augmented inverse probability weighting (average treatment effect: 0.191; 95% CI 0.081–0.300; p = 0.001). No statistically significant associations were observed in in-hospital mortality or the impact of preoperative withholding versus continuation strategies on clinical outcomes.

**Conclusion:**

This study suggests preoperative ACEI/ARB use may be associated with an increased incidence of stage 2 or 3 AKI. While differences in in-hospital mortality and preoperative withholding versus continuation strategies did not reach statistical significance, these findings reveal clinical trends that warrant further investigation.

## Background

Patients undergoing cardiac surgery frequently develop cardiac surgery-associated Acute Kidney Injury (AKI), with an incidence that is up to 30%.^1^ This is associated to a higher risk of morbidity¸ renal replacement therapy requirement, mortality, and delays of hospital discharge.^1^ There have been previous attempts to calculate cardiac surgery-associated AKI risk in these patients. The Cleveland score was validated to identify patients undergoing cardiac surgery who may be at risk for AKI.^2^, ^3^, ^4^ The etiology of this cardiac surgery-associated AKI is multifactorial, systemic inflammatory response syndrome secondary to surgery-induced stress,^5^^,^^6^ and shifting fluid volumes that can lead to fluid overload.^7^

Moreover, these patients usually take drugs that may worsen renal function, such as Angiotensin-Converting Enzyme Inhibitors or Angiotensin Receptor Blockers (ACEI/ARB). Whether the use of ACEI/ARB is independently associated with an increased risk of cardiac surgery-associated AKI needs to be clarified.^8^ 2024 EACTS guidelines on perioperative medication in adult cardiac surgery suggested that continuing ACEI and ARBs should be considered until open-heart surgery,^9^ based on observational retrospective data on low-risk patients undergoing cardiac surgery.^10^^,^^11^

Prior studies on the risk/benefit of ACEI/ARB before cardiac surgery reported conflicting results.^8^^,^^12^, ^13^, ^14^, ^15^, ^16^, ^17^, ^18^, ^19^, ^20^, ^21^, ^22^, ^23^, ^24^, ^25^

Some have recommended maintaining both ACEI or ARB because, during Cardiopulmonary Bypass (CPB), the Renin-Angiotensin-Aldosterone System (RAAS) is activated, potentially leading to renal hypoperfusion. Therefore, continued administration of ACEI and ARB might attenuate this RAAS activation and prevent renal medullary ischemia induced by angiotensin II.^8^^,^^12^, ^13^, ^14^, ^15^, ^16^, ^17^ Furthermore, withdrawing ACEI/ARB may also precipitate acute heart failure and increase the risk of postoperative hypertension.^19^^,^^20^

On the other hand, others have found that ACEI/ARB may be associated with an increased risk of cardiac surgery-associated AKI secondary to a higher risk of hypotension and vasogenic shock,^18^ leading to renal hypoperfusion and a reduced Glomerular Filtration Rate (GFR) due to dilatation of the efferent glomerular arteriole.^21^, ^22^, ^23^, ^24^, ^25^

We hypothesized that preoperative ACEI/ARB use confers an increased risk of postoperative AKI. Accordingly, we sought to evaluate the association between the preoperative ACEI/ARB use and the incidence of stage 2 or 3 AKI as our primary endpoint. Secondary objectives included an assessment of in-hospital mortality, alongside exploratory analyses within the user cohort to elucidate whether withholding versus continuing therapy is associated with the incidence of stage 2 or 3 AKI and in-hospital mortality.

## Methods

### Study design, setting and participants

This prospective multicenter cohort study included high-risk cardiac surgery patients with Cleveland Clinic Score^2^ (CCS) ≥ 4 and aged ≥ 18 years from 14 centers in Spain and the United Kingdom, recruited between July and December 2017. Patients undergoing heart transplantation and pericardiectomy were excluded, as were those with severe preoperative renal insufficiency, defined as need for Renal Replacement Therapy (RRT) or estimated GFR < 15 mL-min^-1^ 1.73 m^-2^. The study was conducted in accordance with the Declaration of Helsinki and approved by the Institutional Review Board (IRB) (Reference #EPA027/15). The informed consent was obtained from all patients or their proxies prior to surgery. The reporting of this study followed the Strengthening the Reporting of Observational Studies in Epidemiology (STROBE) guidelines.

### Exposure, covariates and outcomes

Patients were classified into groups based on ACEI/ARB use. The baseline severity of the patients was estimated using the CCS. The CCS ranges between 0 (lowest risk) and 17 (highest risk) points. This CCS incorporates the following major risk factors: sex, congestive heart failure, left ventricular ejection fraction < 35% (LVEF < 35%), preoperative use of Intra-Aortic Balloon Pump (IABP), Chronic Obstructive Pulmonary Disease (COPD), insulin-requiring diabetes, previous cardiac surgery, emergency surgery, surgery type (only coronary artery bypass graft, only valve, and other cardiac surgeries), and preoperative creatinine.^4^

The primary outcome was the development of stage 2 or 3 AKI according to KDIGO criteria.^26^ To apply these criteria, both creatinine and urine output were measured at Intensive Care Unit (ICU) admission and at 24h, 48h, 72h, 96h, and 7 days postoperatively. The secondary outcome was in-hospital mortality.

The inter-center variability was addressed using a standardized Case Report Form (CRF) shared by all participating institutions. The standardized CRF was designed to ensure that all clinically significant variables related to AKI in cardiac surgery were strictly defined and captured uniformly across all sites.

### Statistical analysis

A post-hoc power sensitivity analysis was conducted to determine the study's capacity to detect varying effect sizes for the primary event. With a total sample size of 249 and a baseline incidence of 26.1% for stage 2 or 3 AKI (reference group: n = 115), the study achieved a statistical power of 72.1% to detect an OR of 2.0. For larger effect sizes, the power increased to 85.3% (OR = 2.25), 92.9% (OR = 2.5), 96.8% (OR = 2.75), and 98.6% for an OR of 3.0. These results suggest that the study was sufficiently powered to detect moderate-to-large clinical effects in the primary analysis. All other analyses were considered exploratory in nature.

The primary hypothesis of this study was that preoperative ACEI/ARB therapy was associated with an increased risk of AKI. Consequently, the primary analysis evaluated the association between ACEI/ARB use and the incidence of stage 2 or 3 AKI. Secondary analyses included the evaluation of in-hospital mortality between ACEI/ARB users and non-users. Furthermore, exploratory secondary analyses were conducted within the ACEI/ARB user group to compare the impact of preoperative withholding versus continuation strategies on both stage 2 or 3 AKI and in-hospital mortality.

Continuous variables were expressed as mean and Standard Deviation (SD), with comparisons performed using the Student’s *t*-test. Categorical variables were presented as frequencies (%) and analyzed using the Chi-Squared test. Baseline balance between groups was assessed using Absolute Standardized Mean Differences (ASMD). The association between treatment exposure and clinical outcomes was first evaluated using a multivariable logistic regression model. Results were reported as Odds Ratios (OR) with corresponding 95% Confidence Intervals (95% CI). The adjusted Risk Difference (RD) and 95% CI was calculated. For clinical interpretation of significant effects, the Number Needed to Harm (NNH) was determined. To address potential confounding by indication and provide a robust framework for causal inference, we further used Augmented Inverse-Probability Weighting (AIPW). This doubly robust estimator was used to calculate the Average Treatment Effect (ATE). To assess the robustness of the observed point estimates against unmeasured confounding in statistically significant associations, the E-value was calculated for both the estimate and the limit of the confidence interval closest to the null. All tests were two-sided, and p-values < 0.05 were considered to indicate statistical significance. All statistical analyses were performed using Stata 19 (StataCorp. 2025. Stata Statistical Software: Release 19. College Station, TX: StataCorp LLC).

## Results

**Figure 1:**
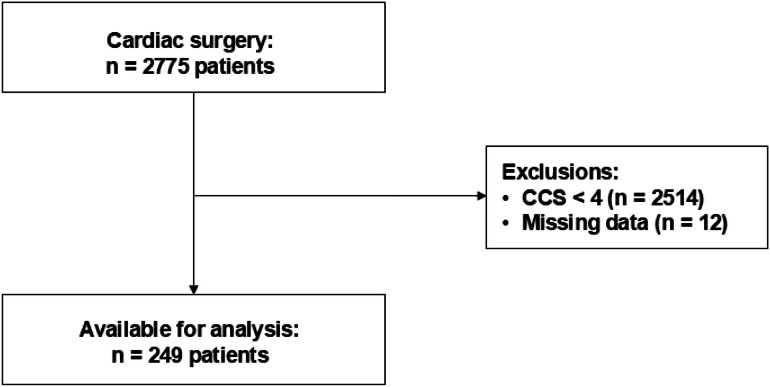
**Study flow diagram.** CCS, Cleveland Clinic Score. The diagram illustrates the selection process of patients from the initial screening to the final analytical sample. Reasons for exclusion are provided according to STROBE guidelines. The CCS is based on ten major risk factors: sex, congestive heart failure, left ventricular ejection fraction < 35%, preoperative use of intra-aortic balloon pump, chronic obstructive pulmonary disease, insulin-requiring diabetes, previous cardiac surgery, emergency surgery, surgery type, and preoperative creatinine.

The patient selection process is illustrated in Figure 1. From an initial screening of 2,775 cardiac surgery patients, 261 met the eligibility criteria. Data completeness was generally high. Missing data for the variables of interest were minimal, limited to ACEI/ARB use (3.5%) and LVEF < 35% (1.2%), the latter being the only severity-defining variable with missing values. Out of 261 eligible patients, 12 (4.6%) were excluded due to missing data, leaving 249 patients for the final analysis.

The baseline demographic and clinical characteristics of the study population are summarized in Table 1, categorized by ACEI/ARB use. A total of 134 patients (53.8%) were ACEI/ARB users. The cohort had an overall mean age of 69.5 years (SD = 11.1), with a predominant representation of white patients (98.4%) and a moderate proportion of women (39%). On average, patients in the ACEI/ARB group were slightly older (71.2, SD: 12.4 vs. 67.5, SD = 12.4 years; AMSD = 0.341; p = 0.009) and showed a higher prevalence of hypertension (90.3% vs. 73.9%; AMSD = 0.438; p = 0.001), LVEF < 35% (14.9% vs. 6.1%; AMSD = 0.291, p = 0.025), and COPD (35.8% vs. 22.6%; AMSD = 0.294; p = 0.023) compared to non-users. The prevalence of CKD (stage III‒V) was high and balanced between groups (77.1%). The patients receiving ACEI/ARB were more likely to undergo elective surgery (73.1% vs. 59.1%; AMSD = 0.299; p = 0.019) and had shorter mean CBP time (124.7, SD: 67.9 vs. 144.8, SD = 71.5 minutes; AMSD = 0.288; p = 0.024). The ACEI/ARB group presented a lower mean CCS compared to non-users (6.0, SD: 1.5 vs. 6.4, SD = 1.5 years; AMSD = 0.255; p = 0.046). No significant differences were observed in recorded preoperative covariates, including hemoglobin levels and GFR, across both groups. The intraoperative management was comparable between groups, including RBC transfusion rates (61%) and the use of vasopressors or inotropes.

**Table 1:** Characteristics of the study population.

	All patients (n = 249)	No ACEI/ARB use (n = 115)	ACEI/ARB use (n = 134)	ASMD	p-value
**Age (years)**	69.5 (11.1)	67.5 (12.4)	71.2 (9.6)	0.341	0.009
**Women, n (%)**	97 (39.0)	40 (34.8)	57 (42.5)	0.160	0.211
**Skin color, n (%)**					
White	245 (98.4)	114 (99.1)	131 (97.98)	0.111	0.626
Black	4 (1.6)	1 (0.9)	3 (2.2)		
**BMI (**k**g.m^-2^)**	27.9 (4.9)	27.3 (4.5)	28.5 (5.1)	0.265	0.039
**Preoperative hemoglobin, g.dL^-1^**	12.1 (2.0)	11.9 (2.0)	12.3 (2.0)	0.200	0.117
**Preoperative comorbid disease, n (%)**					
Hypertension	206 (82.7)	85 (73.9)	121 (90.3)	0.438	0.001
Insulin-requiring diabetes	55 (22.1)	26 (22.6)	29 (21.6)	0.023	0.855
CHF	140 (56.2)	66 (57.4)	74 (55.2)	0.044	0.731
LVEF < 35%	27 (10.8)	7 (6.1)	20 (14.9)	0.291	0.025
Previous cardiac surgery	53 (21.3)	28 (24.4)	25 (18.7)	0.138	0.274
COPD	74 (29.7)	26 (22.6)	48 (35.8)	0.294	0.023
Chronic kidney disease stage III‒V	192 (77.1)	89 (77.4)	103 (76.9)	0.013	0.922
Serum creatinine, mg.dL^-1^	1.5 (0.6)	1.6 (0.7)	1.5 (0.5)	0.153	0.234
eGFR, mL.min^-1^ per 1.73 m^2^	48.1 (20.9)	48.7 (22.2)	47.5 (19.9)	0.055	0.665
Serum uric acid, mg.dL^-1^	7.0 (1.8)	6.9 (1.8)	7.1 (1.8)	0.136	0.286
Preoperative use of IABP, n (%)	18 (7.2)	9 (7.8)	9 (6.7)	0.043	0.736
**Type of surgery, n (%)**					
CABG	27 (10.8)	11 (9.6)	16 (11.9)	0.043	0.781
Valve	72 (28.9)	35 (30.4)	37 (27.6)		
Other cardiac surgeries	150 (60.2)	69 (60)	81 (60.5)		
**Timing, n (%)**					
Elective surgery	166 (66.7)	68 (59.1)	98 (73.1)	0.299	0.019
Urgent / Emergency surgery	83 (33.3)	47 (40.9)	36 (26.9)		
**CCS**	6.2 (1.5)	6.4 (1.5)	6.0 (1.5)	0.255	0.046
**Cross clamp time (minutes)**	96.9 (52.0)	104.1 (55.5)	90.7 (48.1)	0.257	0.043
**CPB time (minutes)**	134.0 (70.2)	144.8 (71.5)	124.7 (67.9)	0.288	0.024
**Intraoperative RBC transfusion, n (%)**	152 (61.0)	70 (60.9)	82 (61.2)	< 0.001	0.958
**Intraoperative RBC transfusion (units)**	1.7 (1.9)	1.7 (2.0)	1.7 (1.9)	0.018	0.886
**Nadir hemoglobin, g.dL^-1^**	7.9 (1.4)	7.8 (1.4)	8.1 (1.3)	0.205	0.116
**Intraoperative dobutamine, n (%)**	119 (47.8)	54 (47.0)	65 (48.5)	0.087	0.807
**Intraoperative norepinephrine, n (%)**	170 (68.3)	81 (70.4)	89 (66.4)	0.031	0.497

Data are presented as mean (standard deviation) unless otherwise stated. ASMD, Absolute Standardized Mean Difference; ACEI/ARB, Angiotensin-Converting Enzyme Inhibitors and Angiotensin Receptor Blockers; BMI, Body Mass Index; CABG, Coronary Artery Bypass Graft; CCS, Cleveland Clinic Score; CHF, Congestive Heart Failure; COPD, Chronic Obstructive Pulmonary Disease; CPB, Cardiopulmonary Bypass; eGFR, estimated Glomerular Filtration Rate; IABP, Intra-Aortic Balloon Pump; LVEF < 35%, Left Ventricular Ejection Fraction < 35%; RBC, Red Blood Cell.

### Association of ACEI/ARB use with the risk of cardiac surgery-associated stage 2 or 3 AKI and in-hospital mortality

The overall incidence of stage 2 or 3 AKI was 32.9%. Specifically, 28.1% of patients developed Stage 1, 12.8% Stage 2, and 20.1% Stage 3 AKI, with 15.7% requiring RRT. Among ACEI/ARB users, 52 out of 134 (38.8%) developed stage 2 or 3 AKI, compared to 30 out of 115 (26.1%) in non-users. The multivariable logistic regression model showed a significant association between ACEI/ARB and stage 2 or 3 AKI following cardiac surgery (OR = 2.79; 95% CI 1.47–5.30; p = 0.002) after adjusting for age, hypertension, LVEF < 35%, COPD, preoperative hemoglobin, surgical urgency and CPB time. In terms of absolute impact, the adjusted RD was 0.188 (95% CI 0.079–0.298; p = 0.001), which corresponds to a NNH of 5.3 (95% CI 3.4–12.7). To assess the potential impact of unmeasured confounding, our sensitivity analysis yielded an E-value of 2.73 for the point estimate (with a lower limit of 1.72). This suggests that while the observed effect is robust to weak confounders, a moderately strong unmeasured factor could potentially render the results non-significant. These findings remained consistent in sensitivity analyses using AIPW. The estimated ATE was 0.191 (95% CI 0.081–0.300; p = 0.001), further supporting the initial multivariable logistic regression findings. After weighting, satisfactory balance was achieved, with all ASMD below 0.1 (Supplementary Fig. 1).

**Figure 2:**
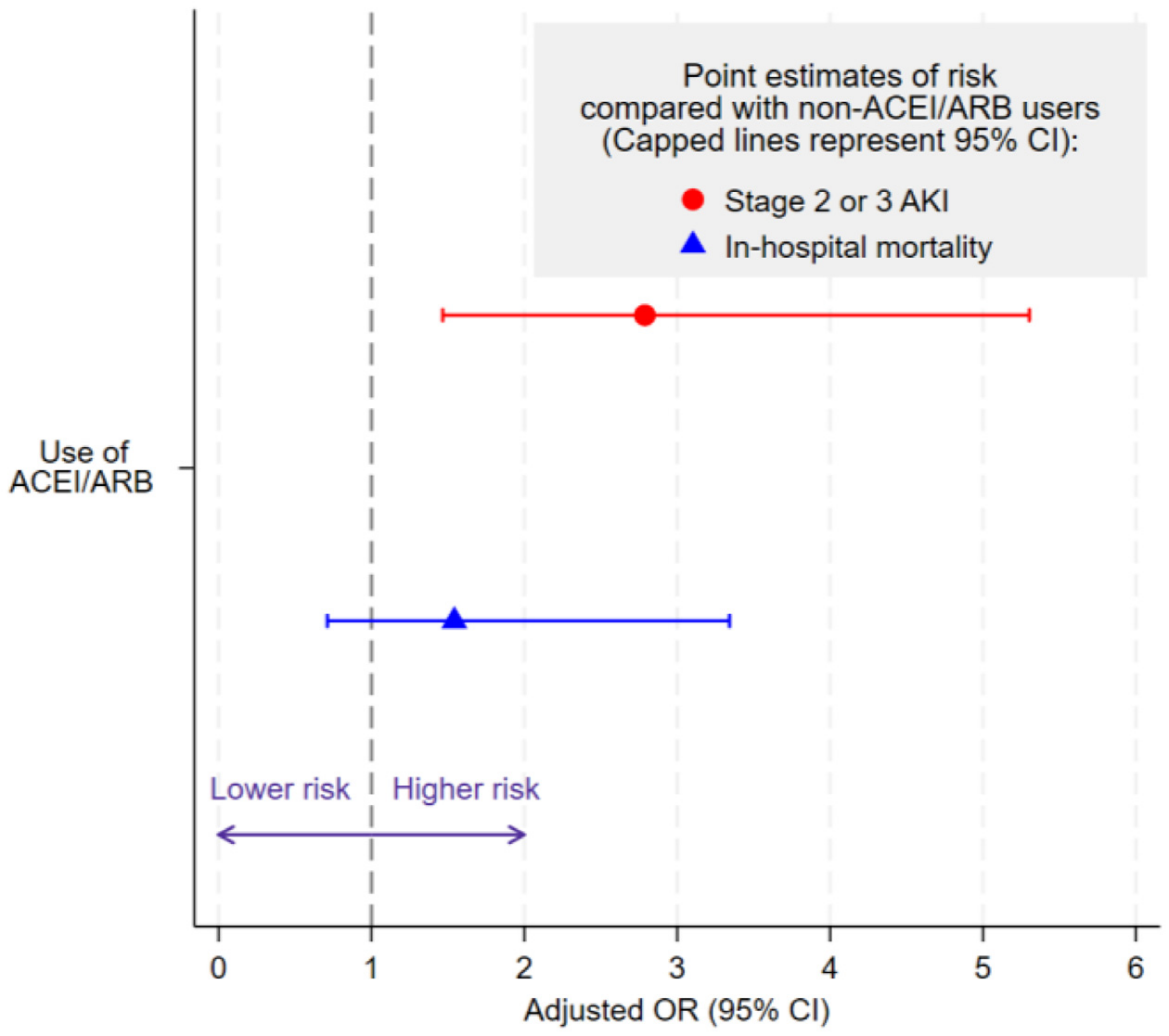
**Risk of cardiac surgery-associated stage 2 or 3 AKI and in-hospital mortality according to the use of ACEI/ARB.** 95% CI, 95% Confidence Interval; ACEI/ARB, Angiotensin-Converting Enzyme Inhibitors and Angiotensin Receptor Blockers; AKI, Acute Kidney Injury; OR, Odds Ratio.

The incidence of in-hospital mortality was 18.7% in the ACEI/ARB group compared with 13.9% in the non-users group, representing a non-statistically significant trend toward higher mortality among ACEI/ARB users (adjusted OR = 1.54; 95% CI 0.71–3.34; p = 0.272) (Fig. 2). The ATE for in-hospital mortality estimated using AIPW remained consistent with this initial observation (ATE = 0.054; 95% CI -0.049–0.156; p = 0.306).

### Clinical outcomes of ACEI/ARB withholding 48 hours prior to the surgery compared with continuation until the day before surgery

The 134 ACEI/ARB users were analyzed to explore the impact of the preoperative ACEI/ARB management strategies (65 patients in the continuing group and 69 patients in the withholding group). The incidence of Stage 2 or 3 AKI was 40.0% in the continuing group compared to 37.7% in the withholding group. After adjustment for age, preoperative hemoglobin levels, hypertension status, LVEF < 35%, chronic obstructive pulmonary disease, surgical urgency, and cardiopulmonary bypass time, withholding treatment was not associated with a significant reduction in the risk of AKI (adjusted OR = 0.68; 95% CI 0.30–1.51). Furthermore, the ATE showed a non-significant risk difference of -8.3 percentage points (95% CI -23.7% to 7.2%; p = 0.293). These results provide a hypothesis-generating signal suggesting a potential benefit of ACEI/ARB discontinuation 48 hours before surgery (Table 2).

**Table 2:** Association between preoperative ACEI/ARB management strategies and postoperative stage 2 or 3 acute kidney injury.

	n	Stage 2 or 3 AKI (%)	Crude OR (95% CI)	Adjusted OR (95% CI)	ATE	p-value
Continuing	65	40	Reference	Reference	−0.083 (−0.237 – 0.072)	0.293
Withholding	69	37.7	0.91 (0.45 – 1.82)	0.68 (0.30 – 1.51)		

95% CI, 95% Confidence Interval; AKI, Acute Kidney Injury; ATE, Average Treatment Effect. Continuing was defined as treatment until the day before surgery. Withholding was defined as cessation 48 hours or more before surgery. The OR derived from logistic regression models. The multivariable logistic regression model was adjusted for age, preoperative hemoglobin levels, hypertension status, left ventricular ejection fraction < 35%, chronic obstructive pulmonary disease, surgical urgency, and cardiopulmonary bypass time. The ATE (risk difference) was estimated using an adjusted augmented inverse probability weighting model. The p-value refers to the ATE estimate.

Similarly, in-hospital mortality was lower in the withholding group (15.9% vs. 21.5%), although this difference did not reach statistical significance (adjusted OR = 0.78; 95% CI 0.29–2.07). The ATE for mortality was -3.4 percentage points (95% CI -15.3%–8.6%; p = 0.581). Consistent with the AKI findings, these results suggest a potential, albeit non-significant, trend toward improved clinical outcomes with preoperative ACEI/ARB discontinuation (Table 3).

**Table 3:** Association between preoperative ACEI/ARB management strategies and in-hospital mortality.

	n	In-hospital mortality (%)	Crude OR (95% CI)	Adjusted OR (95% CI)	ATE	p-value
Continuing	65	21.5	Reference	Reference	−0.034 (−0.153 – 0.086)	0.581
Withholding	69	15.9	0.69 (0.29 – 1.66)	0.78 (0.29 – 2.07)		

95% CI, 95% Confidence Interval; ATE, Average Treatment Effect. Continuing was defined as treatment until the day before surgery. Withholding was defined as cessation 48 hours or more before surgery. The OR derived from logistic regression models. The multivariable logistic regression model was adjusted for age, preoperative hemoglobin levels, hypertension status, left ventricular ejection fraction < 35%, chronic obstructive pulmonary disease, surgical urgency, and cardiopulmonary bypass time. The ATE (risk difference) was estimated using an adjusted augmented inverse probability weighting model. The p-value refers to the ATE estimate.

## Discussion

This study suggests a potential association between ACEI/ARB use and a greater likelihood of cardiac surgery-associated stage 2 or 3 AKI in high-risk cardiac surgery patients. While withholding ACEI/ARB for 48 hours prior to surgery may offer some potential benefits over continuing ACEI/ARB until the day before surgery, the statistical significance was lacking, and so further studies are needed to confirm this association and establish its clinical relevance. These results are consistent with some existing evidence and contribute to clarifying controversial findings in the literature.

These findings are consistent with previous studies regarding the association between cardiac surgery-associated AKI and the use of ACEI/ARB vs. nonusers.^13^^,^^23^^,^^24^^,^^27^

A metanalysis of 69,027 patients from four prospective trials and 25 retrospective observational studies found that preoperative RAS-blocker use was associated with an increased incidence of postoperative AKI (OR = 1.17; 95% CI 1.01‒1.36; p = 0.04) and mortality (OR = 1.20; 95% CI 1.06‒1.35; p = 0.005).^23^ A prospective cohort study of 1,594 adults undergoing cardiac surgery found that ACEI/ARB use was associated with functional AKI in patients who had no treatment (31%), discontinued treatment (34%), or continued treatment (42%; p for trend 0.03). However, it was not associated with structural AKI (neutrophil gelatinase-associated lipocalin, kidney injury molecule-1, interleukin-18, or liver fatty acid-binding protein).^27^ Furthermore, data from a meta-analysis of thirteen studies, 3 Randomized Clinical Trials (RCT) and 10 observational studies including 31,390 patients, found that the use of ACEI was associated with an increase risk of hypotension and postoperative renal dysfunction, but had no significant impact on the risk of early mortality in patients undergoing on-pump CABG.^24^ The hypothesis about the mechanism which might be involved in the increase risk of AKI after cardiac surgery is that ACEI/ARB may cause dilatation of the efferent glomerular arteriole leading to a reduced GFR, as well as, a higher risk of hypotension and vasogenic shock leading to renal hypoperfusion.^13^^,^^18^^,^^21^, ^22^, ^23^, ^24^

In contrast, several other observational studies showed that the preoperative use of ACEI is not associated to AKI after cardiac surgery.^8^^,^^10^^,^^28^ A large-scale meta-analysis of 76,321 patients (including 2 RCT plus 21 cohort studies) showed no association between AKI and patients who received perioperative Renin-Angiotensin System Inhibitors (RASI) preoperatively compared to those who did not receive them.^8^ However, the heterogeneity in this study was relatively high. Most of the included studies were cohort studies (only 2 studies were RCT). A large retrospective cohort study also observed that the use of ACEI was not associated with AKI and was associated with less mortality after cardiac surgery.^10^ However, the retrospective design of this study involves several limitations. Another recent observational retrospective cohort study, including propensity score match analysis, also observed that the use of preoperative ACEI within 48h of Coronary Artery Bypass Graft (CABG) surgery was associated with a decrease in AKI and early mortality at 30 days after CABG. However, patients in this cohort were significantly lower-risk patients for developing AKI than our cohort (overall AKI 6.6% vs. 58.6%).^11^

Our findings regarding “withholding vs maintenance” of ACEI before cardiac surgery are also consistent with previous studies.^29^, ^30^, ^31^ A multicentric observational study on 1,072 patients undergoing cardiac surgery showed that the maintenance of ACEI compared to withholding ACEI 24h before surgery was not associated to AKI after adjusting by propensity matching.^29^ A RCT of 121 patients under current treatment with ACEI/ARB and undergoing cardiac surgery demonstrated no between-group differences in the incidence of cardiac surgery-associated AKI (1.7% vs. 1.6%, p = 0.991) between patients who kept taking their ACEI/ARB until the day of surgery and those who discontinued 48 hours before surgery.^30^ A meta-analysis of 1,663 patients from three RCTs and three prospective cohort studies found no significant between-group difference between patients who continued therapy versus those who discontinued their ACEI/ARB 24‒48h before cardiac surgery (RR = 1.17, 95% CI 0.99–1.38).^31^ Pigott et al. carried out a RCT with 40 patients undergoing CABG that were assigned to either discontinue or continue their ACEI medication until the day before surgery. Notably, the patients who discontinued ACEI in this study were more likely to have hypertension and required less vasopressors and more vasodilators for blood pressure control.^19^ The optimal timing for withholding ACEI/ARB is not clear. Future research could investigate whether longer time (4 days or higher vs. 24‒48h) is required for withholding ACEI before cardiac surgery.

Our results can be also interpreted within the framework of the ACC/AHA 2024 guidelines for perioperative cardiovascular management for non-cardiac surgery, which recommend an individualized approach to perioperative ACE inhibitor and ARB management based on the indication for therapy and surgical risk. On one hand, for patients taking ACEIs/ARBs for hypertension, omission 24 hours before surgery may be beneficial to limit intraoperative hypotension (Class 2b, Level B-R). On the other hand, for patients with an indication for Heart Failure with reduced Ejection Fraction (HFrEF), perioperative continuation is reasonable (Class 2a, Level C-EO).^32^

An important consideration is that patients with high (SBP > 160 mmHg) or low (SBP < 105 mmHg) blood pressure are excluded from RCTs, and enrollment of high-risk patients, including those with HFrEF, has been limited.^33^

The most recent and largest RCT on the use of ACEIs or ARBs in non-cardiac surgery, published after the ACC/AHA 2024 guidelines, showed similar rates of all-cause mortality and major postoperative complications. However, patients randomized to the continuation strategy had more episodes of intraoperative hypotension, and these episodes lasted longer.^33^ Furthermore, a posterior 2025 meta-analysis of five RCTs on 10,773 patients confirmed that discontinuation of ACEIs/ARB likely reduces perioperative hypotension but suggested little to no difference in major clinical outcomes, including AKI, AHF, MI, stroke, and arrhythmias.^34^

Regarding the timing for resuming these agents postoperatively, some observational data suggest that withholding these medications for more than 48h is associated with increased mortality risk in non-cardiac surgery.^35^ However, in cardiac surgery, withholding these medications for 48h postoperatively is part of the KDIGO bundle to reduce AKI risk, as shown in several RCTs in cardiac surgery.^36^^,^^37^ This patient-centered rationale, which also emphasizes communication with anesthesiologists when favoring continuation, could similarly apply to high-risk cardiac surgery patients, given the shared concerns for hemodynamic instability and the need to balance short-term risks against long-term benefits for underlying conditions.

Our study has some limitations that should be considered. While our findings provide evidence for high-risk patients (CCS ≥ 4), their extrapolation to lower-risk groups warrants cautious interpretation. Significant baseline imbalances persisted despite the selection of a homogeneous cohort of high-risk patients. To account for these differences, a multivariable adjustment for relevant covariates was performed to minimize residual confounding. Additionally, AIPW was used as a doubly robust estimation method to address potential confounding by indication. As with most observational studies, we cannot exclude the possibility of residual unmeasured confounding. Specifically, the study lacks data on several clinically meaningful variables, such as Major Adverse Kidney Events (MAKE) at 30 and 90 days (including persistent AKI at 30 or 90 days), intraoperative Mean Arterial Pressure (MAP) nadir, fluid balance, glycemic control, and nephrotoxic exposure. Furthermore, information regarding specific ACEI or ARB agents, including dose, duration, and indication, was not available. Finally, the preoperative ACEI/ARB management strategy decision was clinician driven. This reflects ongoing clinical controversy and may also introduce confounding by indication, as unstable patients are more susceptible to treatment interruption, potentially biasing results in favor of the continuation group. Center-level clustering was not accounted for, leaving potential residual confounding from institutional variability.

The study’s strengths include its prospective design and the use of pragmatic, real-world data from a high-risk cardiac surgery population. Methodological rigor is further underpinned by the adoption of a consensus AKI definition based on Kidney Disease: Improving Global Outcomes (KDIGO) criteria, while an international multicenter collaboration across 14 cardiothoracic hospitals reinforces broad generalizability.

Future research could investigate the optimal timing for withholding ACEI/ARB in different patient subgroups and explore the potential benefits and risks of this strategy. Randomized controlled trials would be valuable for providing definitive evidence on the impact of ACEI/ARB withholding on cardiac surgery-associated AKI outcomes.

## Conclusion

In this multicenter prospective cohort study of high-risk cardiac surgery patients, we observed an association between preoperative ACEI/ARB use and a higher incidence of stage 2 or 3 AKI. Further research is warranted to provide evidence-based guidelines for the management of these patients.

## Data availability statement

The datasets generated and/or analyzed during the current study are available from the corresponding author upon reasonable request.

## AI assistance disclosure

Selected paragraphs throughout the manuscript were revised for linguistic polishing using Google AI Mode (Gemini). All AI-generated suggestions were critically reviewed and verified by the authors, who take full responsibility for the final content.

## Funding

This work was funded by departmental resources (Department of Anesthesiology & Critical Care Medicine. Clínica Universidad de Navarra, Pamplona, Spain).

## Conflicts of interest

The authors declare no have conflicts of interest.

## Appendix Supplementary materials

Image, application 1

## Footnotes

Supplementary material associated with this article can be found in the online version at doi:10.1016/j.bjane.2026.844786.
