# Supplementary material for: Preoperative angiotensin-converting enzyme inhibitor or angiotensin receptor blocker use and acute kidney injury after high-risk cardiac surgery: a multicenter prospective cohort study
Source: Braz J Anesthesiol. 2026 Jul 1;76(4):844786. doi: 10.1016/j.bjane.2026.844786 (PMC13380198; doi:10.1016/j.bjane.2026.844786)

**BJAN-D-25-00662_Supplementary Material**

**Supplementary Figure 1 Absolute standardized mean differences before and after weighting.** COPD, Chronic Obstructive Pulmonary Disease; LVEF < 35%, Left Ventricular Ejection Fraction < 35%.


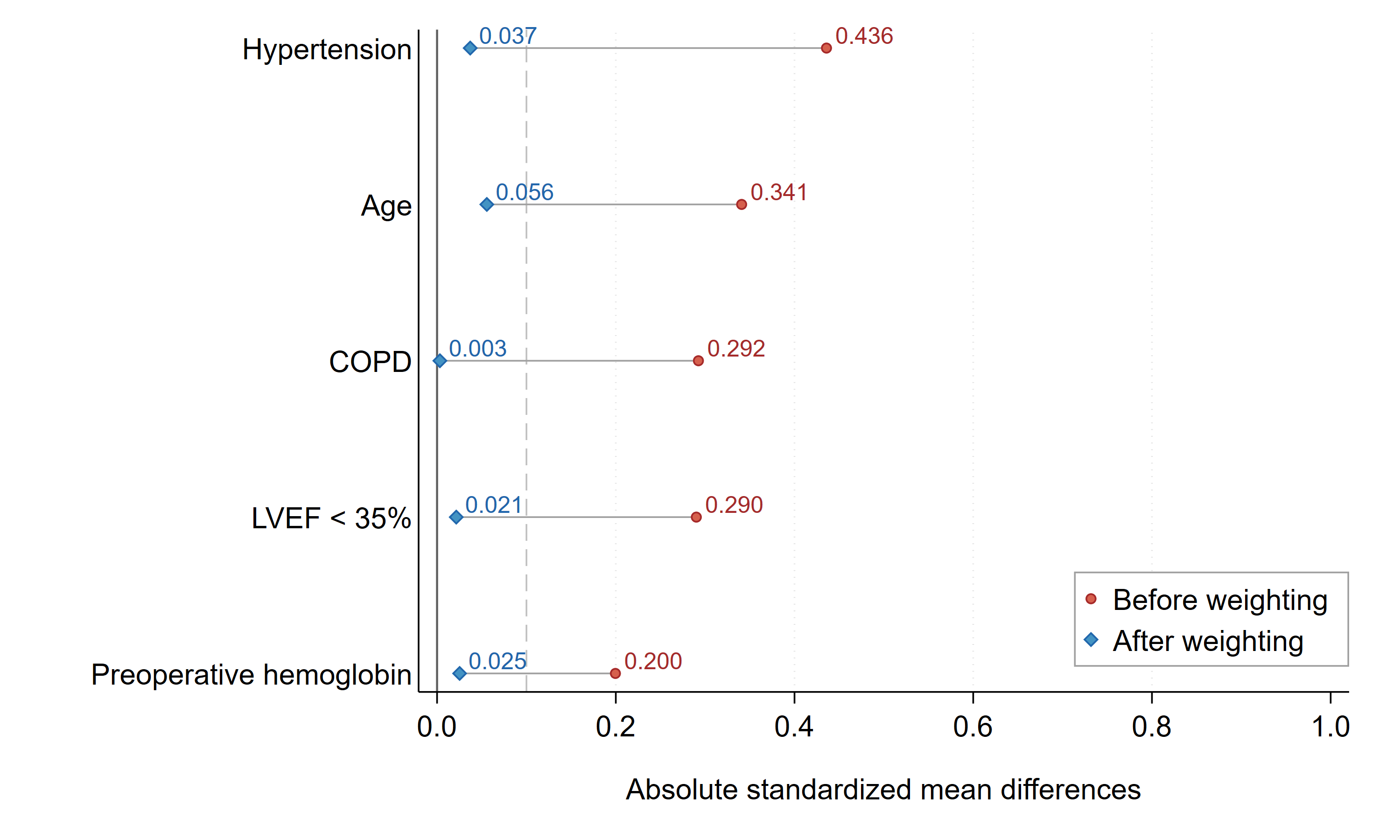

Supplement: Supplementary file 1 [file mmc1.docx]
